# A Call to Include Severe Combined Immunodeficiency in Newborn Screening Program

**DOI:** 10.5041/RMMJ.10135

**Published:** 2014-01-21

**Authors:** Raz Somech, Amos Etzioni

**Affiliations:** 1 Pediatric Department B North, Pediatric Immunology Service, Jeffrey Modell Foundation (JMF) Center, Edmond and Lily Safra Children’s Hospital, Sheba Medical Center, Tel Hashomer, affiliated to the Sackler Faculty of Medicine, Tel Aviv University, Tel Aviv, Israel;; 2 Meyer Children’s Hospital, Rappaport Faculty of Medicine, The Technion – Israel Institute of Technology, Haifa, Israel

**Keywords:** Guthrie cards, immunodeficiency, kappa-deleting recombination excision circles (KRECs), neonatal screening, severe combined immunodeficiency (SCID), T cell receptor excision circles (TRECs)

## Abstract

Quantification of the T cell receptor excision circles (TRECs) has recently emerged as a useful non-invasive clinical and research tool to investigate thymic activity. It allows the identification of T cell production by the thymus. Quantification of TREC copies has recently been implemented as the preferred test to screen neonates with severe combined immunodeficiency (SCID) or significant lymphopenia. Neonatal genetic screening for SCID is highly important in countries with high rates of consanguinous marriages, such as Israel, and can be used for early diagnosis, enabling prompt therapeutic intervention that will save lives and improve the outcome of these patients. TREC measurement is also applicable in clinical settings where T cell immunity is involved, including any T cell immunodeficiencies, HIV infection, the aging process, autoimmune diseases, and immune reconstitution after bone marrow transplantation.

**TAKE-HOME MESSAGES:**

- Severe combined immunodeficiency, a life-threatening condition, can be detected by neonatal screening.
- The earlier the detection and the quicker the implementation of appropriate treatment, the greater the likelihood for improved outcome, even cure, for the affected children.
- TRECs and KRECs quantification are useful screening tests for severe T and B cell immunodeficiency and can be used also to evaluate every medical condition involving T and B cell immunity.

## INTRODUCTION

In 1958, a 15-month-old girl was diagnosed with phenylketonuria (PKU), a potentially life-threatening metabolic disease. A few years later, her uncle, Dr Robert Guthrie, an American microbiologist, published his seminal paper on the feasibility of mass screening for PKU, using a bacterial inhibition assay and dried blood spot (DBS) samples. This innovation can be regarded as the birth of newborn screening (NBS). Over the last 50 years, NBS has been an acclaimed success, and many thousands of children have been saved from devastating effects of severe inborn metabolic disorders, congenital endocrinopathies, hemoglobinopathies, and other genetic disorders because of early diagnosis of their conditions. Many countries across the globe have made NBS mandatory.^1^

The expansion of a national NBS panel inevitably presents many scientific, technical, ethical, and policy issues that must be addressed prior to the addition of a new entity to the test panel. In general, the common criteria for including a disease in NBS are that 1) the prevalence of the disease justifies the costs involved; 2) the disorder is not readily identified by means of physical examination; 3) the disease must cause serious medical complications; 4) early diagnosis and treatment of the disease improves prognosis and leads to an acceptable outcome; and 5) the screening methodology is sensitive, specific, economic, validated, and available.^2^

NBS is of utmost importance in counties such as Israel, where the high rates of consanguineous marriages make inherited diseases much more common than in other parts of the world.^3^ For example, genetic disorders, such as congenital hypothyroidism,^4^ glucose-6-phosphate dehydrogenase deficiency,^5^ and PKU,^6^ have been found in a relatively high frequency in both the Israeli Jewish and non-Jewish communities. Thus far, the Israeli NBS program includes PKU, congenital hypothyroidism, congenital adrenal hyperplasia, maple syrup urine disease, homocystinuria, tyrosinemia, methylmalonic acidemia, propionic acidemia, glutaric aciduria, medium- and very-long-chain acyl-CoA dehydrogenase deficiency, and a few other metabolic diseases.

Another important disease that should be considered for inclusion in the Israeli NBS is severe combined immunodeficiency (SCID).^7^ SCID encompasses a heterogeneous group of genetic disorders characterized by thymic dysplasia and arrest in T lymphocyte maturation. There is also variable expression of B and natural killer (NK) cells, and patients are categorized into either SCID with absence of T lymphocytes but presence of B lymphocytes (T-B+ SCID) or SCID with absence of both T and B lymphocytes (T-B- SCID). Regardless of the immunologic phenotype, patients with SCID present with similar clinical features, including early-onset severe respiratory tract infections, chronic diarrhea, and failure to thrive. All affected individuals, without exception, will have a fatal outcome unless the immune system is promptly restored by means of strict isolation and prophylactic antibiotics, followed by hematopoietic stem cell transplantation (HSCT) or gene therapy.^8^ SCIDs have a worldwide prevalence of approximately 1:50,000 live births and are more common in male subjects, reflecting the over-representation of X-linked SCID (γ chain mutation), the most common form of SCID in human subjects. In Israel, the prevalence is expected to be higher, and the most frequent SCID phenotype is the autosomal-recessive T-B- RAG1 or RAG2 mutations, while the X-linked SCID is rare.^9^

## THE RATIONALE TO INCLUDE SCID IN NEWBORN SCREENING

Diagnosing SCID is a pediatric emergency. Affected children will eventually die of disease if appropriate diagnosis and treatment are not instituted. The rationale behind including SCID in an NBS program was based on a number of assumptions^10^:

1. Importance of early diagnosis and immediate provision of life-saving treatment (HSCT); transplantation before the age of 3 months has a 95% success as opposed to 70% later on.
2. Saving the lives of many children diagnosed too late or misdiagnosed.
3. Avoidance of inefficient, costly, and dangerous diagnostic tests.
4. Provision of diagnosis and of advice on reproductive risks to families with genetic risks.
5. Establishment of the incidence and true spectrum of SCID.
6. Overcoming the confounders that over 80% of SCID cases are sporadic and that there may not be a family history or that family history can be missed during evaluation.
7. Avoiding the high cost of prolonged antimicrobial treatment and long hospitalization.

SCID babies diagnosed at birth because of a positive family history were reported to have a significantly improved outcome compared with the first-ever presenting family member.^7^ The overall improved survival of more than 90% is related to a reduced rate of infection and significantly improved transplantation outcome irrespective of donor choice, conditioning regimen used, and underlying genetic diagnosis. Similarly, Chan et al.^11^ reported an infant mortality rate of 42% for 138 neonates who were not tested at birth compared to a 15% mortality rate for 20 neonates who were tested at birth. Moreover, early diagnosis of SCID was also proved to be relatively cost-effective in spite of the low incidence of the disease.^12^ Indeed, a recent systematic review demonstrated the benefits of early treatment of SCID and the feasibility of population-based newborn screening for immunodeficiency.^13^

## SCREENING ASSAYS CONSIDERED FOR SCID NBS

Since typical SCID patients will present with profound lymphopenia due to reduced T cells, a complete blood count with an absolute lymphocyte count (ALC) was proposed for the purposes of SCID newborn screening. During infancy, an ALC count of less than 2,500/μL is potentially pathogenic and requires further evaluation. Then, flow cytometry should be performed to determine the presence of T, B, and NK lymphocytes, the repertoire of the T cell receptors, and the response of the T cells to mitogen or antigen stimulations in order to confirm the diagnosis.^14^ Importantly, none of these aforementioned tests is available on DBSs obtained during NBS. Moreover, many patients may be misdiagnosed by assessment of their ALCs, including those with high numbers of B cells and possibly NK cells or patients with residual, autoreactive (e.g. the Omenn phenotype), or alloreactive (transplacentally acquired maternal cells) T lymphocytes. Thus although it is a valuable and often overlooked clinical tool for individual patients and high-risk settings, the ALC has too much overlap between infants with SCID and healthy infants to be suitable for population-based SCID screening.

Immunoassay platforms for SCID newborn screening have been suggested, including immunoassay with CD3 as a marker for T cells, with CD45 as a marker for total leukocytes,^15^ or the detection of IL-7 for functional T cell immunity.^16^ However, these assays were not sufficiently informative to be considered for widespread screening. Of all the approaches considered for SCID screening, testing for T cell receptor (TCR) excision circles (TRECs), a DNA biomarker of normal T cell development, has proven to be the most successful.

## THE IMMUNOLOGY BEHIND TREC FORMATION

**Figure 1.**
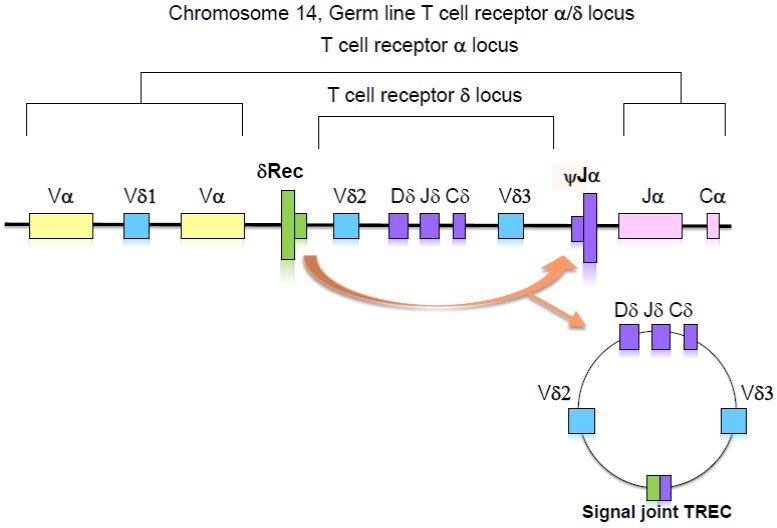
**Formation of TRECs.** The whole TCRD gene is interspersed with TCRA gene segments along chromosome 14q11. The D-locus excision forms an episomal DNA circle with a characteristic signal joint DNA region (the so-called sjTREC).

The thymus gland is the main organ for T cell development and maturation. Inside this gland, the T cells undergo three main processes in order to become immunologically functional after their release to the peripheral blood. Those processes are: 1) the expression of either CD4 or CD8 molecules; 2) random DNA rearrangements of the cell receptor chains to produce a diverse TCR repertoire that will enable the targeting of unlimited numbers of foreign antigens; and 3) elimination of possibly harmful TCRs that may recognize self-antigens and cause autoimmunity. The TCR is composed of disulfide-linked heterodimeric proteins which are composed of either alpha/beta (>95%) or gamma/delta chains. These protein chains have different segments encoded by non-continuous gene segments. The segments are joined in a tightly regulated order during T cell differentiation via the gene rearrangement processes. An extra chromosomal circular excision by-product (TREC) is formed when the signal ends are ligated. The detection of TREC in the peripheral blood is a clear indication of the occurrence of the rearrangement process.^17^ Moreover, TREC levels in human peripheral blood were shown to reflect the nature of thymus activity.^18^ The specific TCRD gene excision (that is interspersed with TCRA gene segments along chromosome 14q11) is widely used to detect thymic activity (Figure 1). This excision reflects a late rearrangement event (when its dilution inside the thymus is minimal) and is common to 80% of the thymocytes. Real-time quantitative polymerase chain reaction (RTqPCR) is the preferred assay for detecting TRECs because it is sensitive and accurate and based on the specific detection of the amplified target sequences during each PCR cycle.^17^

## CLINICAL IMPLICATIONS OF TREC QUANTIFICATION

Because TREC quantification can accurately estimate thymic activity and T cell numbers, it has been proposed for use in several primary or secondary immunodeficiencies where T cell immunity is known to be affected (Table 1). Any routine immunological workup of a patient with SCID or other form of severe T cell lymphopenia should include TREC levels.^14^ It is considered the best screening assay for severe T cell lymphopenia through newborn screening on DBSs (see below). TREC quantification was also suggested in patients with syndromes involving T cell immunity, such as the 22q11.2 deletion syndrome, in order to estimate the degree of their T cell immunity.^19^

**Table 1.** Use of TREC Quantifications in Different Clinical Settings.

**Use of TREC Quantifications**
Evaluating patients with SCID or severe lymphopenia
Assay for neonatal screening for SCID or severe lymphopenia
Assessment of activity of autoimmune disorders
Immune reconstitution after bone marrow transplantations
Normal and abnormal aging processes
Studying normal T cell development
Response to HIV treatment

Aging is a well-described secondary immunodeficiency state. One possible explanation for this association is reduced thymic activity due to age-associated thymic involution.^20^ Therefore, the number of TRECs is suspected to be low in the elderly, mainly due to the peripheral cell division that lowers the TREC content of mature T lymphocytes, but also because of reduced thymic activity.

Assessment of T cell homeostasis in autoimmunity is possible through the parallel detection of TREC levels and TCR clonality.^19^ This explains why decreased TREC levels were found in patients with active autoimmune diseases, such as juvenile idiopathic arthritis, active systemic lupus erythematosus, and primary progressive multiple sclerosis. We used TREC levels to describe the T cell compartment in the synovial fluid in pediatric patients with juvenile idiopathic arthritis. We showed an alteration in the T cells from synovial fluid, which correlated with disease phenotype, assumedly secondary to enhanced proliferation, clonal TCR restriction, and reduced T cell production.^21^

TREC quantification is also used to monitor T cell immune reconstitution after bone marrow transplantation (BMT). Various studies have been performed in order to test immune reconstitution after BMT, and quantification of TRECs and analyses of the TCR repertoire were the most advanced assays used for this purpose. The presence of TRECs early after transplant was found to be the best early marker that may predict the outcome of the BMT procedure.^21^ Following TREC and kappa-deleting recombination excision circle (KREC) levels enabled the monitoring of the kinetics of early T and B cell immune recovery after BMT in RAG2-deficient SCID patients.^22^ We therefore suggested that these assays should be used to monitor outcome and tailor specific therapy for patients undergoing BMT.

HIV infection affects the thymus, causing both its dysfunction and involution. As such, TREC measurements in HIV patients are highly beneficial before and during therapy. HIV-reconstituting children were shown to have a better thymic function than HIV-reconstituting adults, suggesting that increased thymic output could play a predominant role in immune reconstitution, at least in children.^23^ Taken together, these findings indicate that the TREC level appears to be a useful marker of thymic function in HIV-infected patients.

## NBS FOR SCID USING TREC

**Figure 2.**
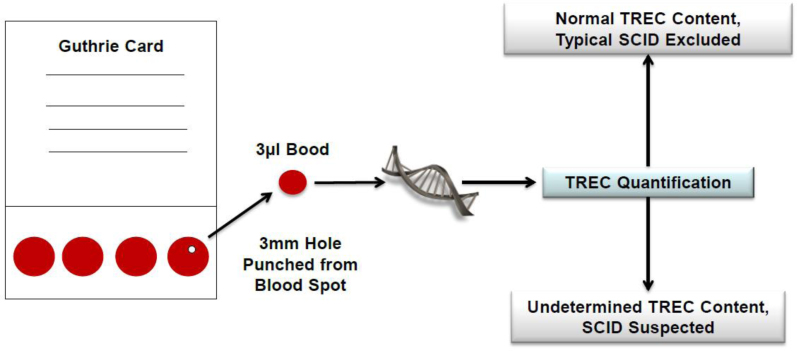
**Neonatal Screening for SCID Using TREC Content.** The Guthrie card, used to collect heel-stick blood, is obtained routinely from every newborn. Quantitative PCR is performed from a 3-mm punch, and the number of TRECs is determined by comparison with standard serial dilutions of a plasmid containing the TREC sequence.

The utility of TREC to identify SCID was first suggested by several pioneering studies which confirmed TREC level accuracy for identifying infants with SCID regardless of genetic cause (Figure 2). Chan and Puck^24^ showed that, unlike filters obtained from normal newborns, which had a mean of 1,020 TRECs in two 3-mm punches, samples obtained from 23 infants with SCID had <30 TRECs. Those authors concluded that TRECs are a stable analyte that can identify T cell lymphopenia in NBS cards. A larger pilot study on 5,766 newborns and positive controls consisting of SCID patients and whole blood specimens selectively depleted of naive T cells revealed similar results.^25^ An important report by Morinishi et al. showed that the TREC assay can also be used in diagnosing atypical SCID patients presenting with maternal T cell engraftment or leaky T cells, including the Omenn phenotype.^26^ No false-positive or -negative results were reported in that study. Three years ago, after seven successful pilot studies conducted in the USA, TREC testing was incorporated as part of the newborn screening panels in several states, including Wisconsin, Massachusetts, and California. It became the first genetic disorder of the immune system to be amenable to NBS. So far, almost one million newborns have been screened, and 14 cases of SCID, 6 cases of SCID variants, and 40 cases of non-SCID immunodeficiency were identified. These results suggest that SCID is more common than initially thought. More importantly, all SCID patients could receive appropriate therapy, and all survived. False-positive results, namely amplification failure of TREC in non-immunodeficient newborns, requiring a second heel stick was reported in <0.08% of examined samples. This failure rate is similar to other newborn screening procedures. False-positive results were found mainly in preterm neonates weighing less than 1,500 g or due to inappropriate performance of the test. An algorithm of how to follow TREC levels in preterm infants was suggested to overcome this limitation. Importantly, in each sample, where TREC is analyzed, a DNA detector is also measured (e.g. actin or RNaseP) to verify the accuracy of the results. Taken together, these results emphasize that screening for SCID is feasible and that SCID is not as rare as commonly believed. TREC assay can identify other T cell lymphopenia, and careful immune evaluation is needed for positive screen results. Finally, implementation of SCID screening will enable the identification of the true incidence of SCID, the causes of many other cases of T cell lymphopenia, and the incidences of SCID and other types of T cell lymphopenia among different ethnicities.

## THE ISRAELI EXPERIENCE OF NBS FOR SCID

The frequency of SCID in Israel can be expected to be higher than in most of Western countries because of the relatively high rate of consanguinity in selected communities. The first national survey in Israel, performed in 2002, identified 39 SCID patients, of whom 20 (51%) were T-B- SCID phenotype and 8 (20%) were T-B+ SCID phenotype.^27^ Nine years later, 14 new cases (T-B- SCID = 6 and Omenn syndrome = 8) were reported, and consanguinity was reported in 50% of the affected families.^28^ Interestingly, eight of the patients who had Omenn phenotype presented with normal numbers of lymphocytes and could therefore have been misdiagnosed if absolute lymphocyte count-based methodology for the diagnosis of SCID had been used. Since the most frequent type of SCID genotype in Israel is the autosomal-recessive T-B- SCID, undetectable B cells in NBS is also very informative for the diagnosis of SCID and can immediately point to the specific abnormal gene (RAGs). This can be easily done simultaneously with TREC detection using quantification of KREC copies. The latter is used for the detection of newly produced bone marrow cells, making it a very sensitive and accurate way to estimate B lymphocytes. We recently assessed TREC and KREC counts to determine their ability to identify patients with combined T and B cell immunodeficiency in Israel.^29^ Seven Israeli children who had been born between 2010 and 2011 and later diagnosed as having SCID were studied. TRECs and KRECs in their peripheral blood upon diagnosis and those in their neonatal Guthrie cards were analyzed using the accepted RTqPCR. The first features suggestive of SCID were presented at a mean age of 3.1 ± 2.4 months in all patients, but the diagnosis was not made until 4.1 ± 2.9 months later. Their TRECs were undetectable or significantly low during their clinical diagnosis and in their originally stored Guthrie cards, irrespective of the amount of their circulating T cells. KRECs were undetectable in the SCID patients who displayed B cell lymphopenia in addition to T cell lymphopenia. These results indicate that the quantification of TRECs is a sensitive and specific screening test for SCID and that the additional quantification of KRECs can screen for B cell lymphopenia. It is quite logical to assume that several more children were not diagnosed; we estimate that every year about seven to eight new cases of SCID are born. Thus the true incidence is about 1/20–25,000 (annual birth number in Israel is around 170,000).

In conclusion, measurement of TREC content has become the best non-invasive clinical and research tool to investigate thymic activity. It allows the identification of recent thymic emigrants in peripheral blood and detection of T cell production by the thymus. It has recently been implemented in several states in the USA as a test to screen neonates for SCID, serving as the most sensitive and specific assay in such a devastating disease. Neonatal genetic screening for SCID, especially in countries with high rates of consanguine marriages such as Israel, can be used for early diagnosis, enabling prompt therapeutic intervention that will save lives and improve the outcome of these patients. TREC measurement is expected to provide much-needed clinical and research information on T cell immune dysfunction and can be used in clinical settings where T cell immunity is involved, including T cell immunodeficiencies, HIV infection, the aging process, autoimmune diseases, and immune reconstitution after BMT.

## Abbreviations:

ALC: absolute lymphocyte count
BMT: bone marrow transplantation
DBS: dried blood spot
HSCT: hematopoietic stem cell transplantation
KRECs: kappa-deleting recombination excision circles
NBS: newborn screening
NK: natural killer
PCR: polymerase chain reaction
PKU: phenylketonuria
RTqPCR: real-time quantitative polymerase chain reaction
SCID: severe combined immunodeficiency
TCR: T cell receptor
TRECs: T cell receptor (TCR) excision circles.
